# My Diabetes Coach, a Mobile App–Based Interactive Conversational Agent to Support Type 2 Diabetes Self-Management: Randomized Effectiveness-Implementation Trial

**DOI:** 10.2196/20322

**Published:** 2020-11-05

**Authors:** Enying Gong, Shaira Baptista, Anthony Russell, Paul Scuffham, Michaela Riddell, Jane Speight, Dominique Bird, Emily Williams, Mojtaba Lotfaliany, Brian Oldenburg

**Affiliations:** 1 School of Population and Global Health The University of Melbourne Melbourne Australia; 2 The Australia Center for Behavioral Research in Diabetes Diabetes Victoria Melbourne Australia; 3 Princess Alexandra Hospital, Queensland Australia; 4 Centre for Health Services Research The University of Queensland Brisbane Australia; 5 Menzies Health Institute Queensland Griffith University Queensland Australia; 6 Kirby Institute University of New South Wales Sydney Australia; 7 School of Psychology Deakin University Geelong Australia; 8 Faculty of Health and Medical Sciences University of Surrey Surrey United Kingdom

**Keywords:** type 2 diabetes mellitus, self-management, health-related quality of life, digital technology, coaching, mobile phone

## Abstract

**Background:**

Delivering self-management support to people with type 2 diabetes mellitus is essential to reduce the health system burden and to empower people with the skills, knowledge, and confidence needed to take an active role in managing their own health.

**Objective:**

This study aims to evaluate the adoption, use, and effectiveness of the My Diabetes Coach (MDC) program, an app-based interactive embodied conversational agent, *Laura*, designed to support diabetes self-management in the home setting over 12 months.

**Methods:**

This randomized controlled trial evaluated both the implementation and effectiveness of the MDC program. Adults with type 2 diabetes in Australia were recruited and randomized to the intervention arm (MDC) or the control arm (usual care). Program use was tracked over 12 months. Coprimary outcomes included changes in glycated hemoglobin (HbA_1c_) and health-related quality of life (HRQoL). Data were assessed at baseline and at 6 and 12 months, and analyzed using linear mixed-effects regression models.

**Results:**

A total of 187 adults with type 2 diabetes (mean 57 years, SD 10 years; 41.7% women) were recruited and randomly allocated to the intervention (n=93) and control (n=94) arms. MDC program users (92/93 participants) completed 1942 chats with *Laura*, averaging 243 min (SD 212) per person over 12 months. Compared with baseline, the mean estimated HbA_1c_ decreased in both arms at 12 months (intervention: 0.33% and control: 0.20%), but the net differences between the two arms in change of HbA_1c_ (−0.04%, 95% CI −0.45 to 0.36; *P*=.83) was not statistically significant. At 12 months, HRQoL utility scores improved in the intervention arm, compared with the control arm (between-arm difference: 0.04, 95% CI 0.00 to 0.07; *P*=.04).

**Conclusions:**

The MDC program was successfully adopted and used by individuals with type 2 diabetes and significantly improved the users’ HRQoL. These findings suggest the potential for wider implementation of technology-enabled conversation-based programs for supporting diabetes self-management. Future studies should focus on strategies to maintain program usage and HbA_1c_ improvement.

**Trial Registration:**

Australia New Zealand Clinical Trials Registry (ACTRN) 12614001229662; https://anzctr.org.au/Trial/Registration/TrialReview.aspx?ACTRN=12614001229662

## Introduction

Type 2 diabetes mellitus (T2DM) is a common chronic condition that places a significant burden on individuals and the health care system. The prevalence of diabetes has risen substantially over the past two decades worldwide [1]. However, clinical outcomes have not improved significantly despite considerable investment and advances in treatments and technologies over this period [2]. Delivering self-management support to people with T2DM is essential to reduce the health system burden and to empower people with the skills, knowledge, and confidence needed to take an active role in managing their own health [3].

Health coaching programs, incorporating continuous feedback and reinforcement [4] delivered to people with diabetes by health care professionals, have been shown to be an effective strategy to improve glycemic management [3,5]. However, the human delivery of such programs on a large scale is very challenging, given the health system and workforce constraints. The emergence of information and communication technologies in recent years offers the potential to deliver such programs at scale and with relatively low costs [6]. Our previous studies and others have demonstrated the effectiveness and cost-effectiveness of using technology-enabled programs, including computer-based or telephone-based coaching, in promoting behavior change and self-management among adults with T2DM [7-9]. The increasing ubiquity of smartphones and other smart devices now offers the potential to further optimize the delivery of such programs at scale by the use of apps.

Despite the increasing use of mobile apps for health purposes, reviews have found that existing digital health solutions are not generally able to meet the needs of people with diabetes, and more evidence is required before their wider scale-up [10,11]. Most commercially available apps employ limited use of behavior change techniques and inadequate features, other than self-monitoring [12,13]. Furthermore, people with diabetes expect apps to be engaging; incorporating multiple functions; and covering a broad range of content, including psychological and emotional support [14]. Although some recent systematic reviews and meta-analyses have indicated a modest effect for app-based interventions to support diabetes self-management [10,11,15], there still remains a great deal of uncertainty about their feasibility, acceptability, and effectiveness in the *real world* [10,15-17].

By adapting our team’s previous effective and cost-effective Telephone-Linked Care for Diabetes (TLC diabetes) program [8] to a more contemporary technology platform, we designed the My Diabetes Coach (MDC) program, which incorporates an app-based interactive embodied conversational agent, *Laura*, to provide more accessible and engaging self-management support, monitoring, and coaching to adults with T2DM in Australia. We undertook a hybrid effectiveness-implementation study [18] with a randomized controlled design. The study aims to evaluate both the implementation of the MDC program over 12 months and its effectiveness. In this paper, we report on the program adoption and use during the trial and program effectiveness in terms of the coprimary outcomes and selected secondary outcomes. The report of the study is in line with the CONSORT (Consolidated Standards of Reporting Trials) guideline and the extension guideline of the CONSORT-eHealth guidelines.

## Methods

### Study Design Overview

This trial is a two-arm, open-label, randomized controlled trial with participants recruited between June 2016 and April 2017 in Australia. The trial was registered before recruitment (Australia New Zealand Clinical Trials Registry ID: ACTRN12614001229662). Full ethics approval was granted by the University of Melbourne’s Human Research Ethics Committee (Ethics ID 1442433). Participants provided written informed consent and returned the informed consent forms to the research team via email or fax, including permission for their general practitioners (GPs) to regularly share clinical data with the study team.

### Study Participants

Adults (aged ≥18 years) diagnosed with T2DM, registered with the National Diabetes Service Scheme (NDSS) for less than 10 years, with basic English language skills, who had access to an internet-enabled smart device with an up-to-date operating system (at least iOS 8.0 for Apple and 4.2 for Android) were eligible to participate in the study. Participants were ineligible if they were pregnant or planning to become pregnant, had severe comorbid conditions that would compromise their participation, or did not have stable doses of diabetes-related medication over the previous 4 weeks or more.

To assist with recruitment, on behalf of the research team, the NDSS sent invitation letters to registered adults with T2DM living in the Australian states of Queensland, Victoria, and Western Australia. These 3 states comprise 54.5% of all Australians with diagnosed diabetes [19]. Recruitment information was also posted on social media via the University of Melbourne website, Facebook, and Twitter and was made available via Diabetes Australia and state-based diabetes organizations (eg, newsletters or magazines). This approach enabled people from all Australian states or territories to enter the study. People who submitted an expression of interest were contacted by the research team by telephone within 48 hours to complete eligibility screening. Eligible participants who provided consent and completed the baseline survey were formally enrolled in the study.

### Randomization

Participants were randomly allocated to the intervention or control arm using a 2x4 block randomization sequence, programmed into a Redcap data management system [20], with the individual as the unit of randomization. The program coordinator informed participants and their GPs of the randomization allocation via telephone or email. Due to the nature of the intervention, participants, their GPs, and the program coordinator could not be blinded to participant study allocation. A statistician who verified the data analysis was blinded to the randomization.

### Intervention and Control

#### Intervention: MDC Program

Participants allocated to the intervention arm received access to the MDC program for up to 12 months. The overall program comprises 5 components: the MDC app; a printed user guide; the MDC website; an optional blood glucose meter with Bluetooth capability (Accu-Chek Aviva Connect, Roche Diabetes Care); and a small number of brief, structured interactions with a program coordinator, primarily for technical assistance. The MDC app was adapted from the previous TLC diabetes program [8] and was co-designed with the support of the Bupa Foundation and a technology company named Clevertar. Multimedia Appendix 1 details the MDC program and these 5 components.

MDC delivers personalized support, monitoring, and motivational coaching via an embodied conversational agent, *Laura*, through a series of modules covering blood glucose monitoring, healthy eating, physical activity, medication taking, and foot care. The conversational scripts and algorithms guiding each individual’s progress were designed by applying behavior change theories and techniques, including the transtheoretical model [21], social cognitive theory [22], gamification [23], and other concepts derived from chronic disease self-management [24]. Algorithms were further tailored according to the clinical targets and recommendations provided by each participant’s GP.

Participants were encouraged to use the app weekly to complete online modules by chatting with *Laura* or touching buttons on the screen. Each *appointment* module with *Laura* began with a review of progress with feedback, education and counseling on the chosen topic, and incorporated tips on overcoming barriers, followed by a short quiz and closing remarks.

In addition to the MDC app, participants were also encouraged to regularly access the user guide and the MDC website and to join the discussion forums on diabetes self-management topics posted fortnightly by the program coordinator on the website. The program coordinator, supported by a web-based user management portal, assisted participants in dealing with system-generated technical alerts by communicating with participants, their GPs, and Clevertar. The program coordinator also telephoned participants after 1, 4, 8, 12, and 24 weeks of program access to answer questions, to troubleshoot technical issues, and to encourage program use.

#### Control Arm

Participants in the control arm were encouraged to continue their routine diabetes self-care, including access to health care services, resources accessed via NDSS, and the diabetes not-for-profit organizations in their states. They received a quarterly project newsletter to maintain their interest in the study. Following the 12-month data collection, participants in the control arm received access to the MDC program if they wished.

### Measurement and Outcomes

We used the Reach, Effectiveness, Adoption, Implementation, and Maintenance framework to evaluate the impact of the MDC intervention, which covered the 5 dimensions in terms of reach, effectiveness, adoption, implementation, and maintenance [25]. This paper focuses on program adoption, use, and effectiveness.

#### Program Adoption and Use

Program adoption and use were tracked using the program management portal. The key metrics included the number of completed chats with *Laura*, the total duration of chats, and the number of blood glucose levels uploaded by participants. Other metrics included the number of technical and clinical alerts generated by the system for the project coordinator and the number of posts on the web-based discussion forum of the MDC website.

#### Program Effectiveness

Program effectiveness was measured by both clinical and psycho-behavioral outcomes. The coprimary outcomes were changes (12 months compared with baseline) in glycated hemoglobin (HbA_1c_) and health-related quality of life (HRQoL), which were examined in terms of between-arm differences. The secondary time point of analysis examined the change between baseline and 6 month. HbA_1c_ (reported as % and mmol/mol) was measured through a pathology blood test that each participant’s GP requested. HRQoL was assessed via participants’ completion of the Assessment of Quality of Life (AQoL)-8D scale, which is a 35-item multi-attribute utility instrument covering 8 dimensions focused on independent living, happiness, mental health, coping, relationships, self-worth, pain, and senses [26,27]. The AQoL-8D provides a utility score ranging from −0.04 to 1, where higher scores indicate better utility value in HRQoL.

Selected secondary outcomes reported in this paper include anxiety and depressive symptoms, assessed by the Hospital Anxiety and Depression Scale (HADS) [28]; diabetes-specific distress, assessed by the Problem Areas in Diabetes scale [29]; and clinical measurement of body weight. Secondary outcomes were assessed by examining between-arm differences at 6 and 12 months compared with baseline. Further secondary outcomes will be reported elsewhere.

### Data Collection Procedures

Data were collected at 4 time points, including screening, baseline, 6 months, and 12 months post randomization. During the screening telephone calls, the research team contacted participants and recorded sociodemographic characteristics (age, sex, education, employment, and language capability), clinical characteristics (duration of NDSS registration and stabilization of the health condition), and app use variables in the REDcap data collection system [20]. Assessments of participants’ clinical and psycho-behavioral data were conducted following a standard protocol at baseline and at 6 and 12 months. At each time point, the research team contacted participants in both arms via email to advise that data collection was required. The full data collection details are listed in Multimedia Appendix 2. The email contained a link to the web-based self-administered questionnaire via REDcap. The research team also requested clinical measures from participants and their GPs. If clinical measures were taken within the past 3 months, GPs provided these to the research team; otherwise, participants made an appointment with their GPs to request new tests. GPs provided the clinical information to the research team via fax, and the research team entered the information into REDcap. Data related to program adoption and use were captured automatically by the program (throughout the study period, for all program users), and the information was provided to the research team by Clevertar.

### Analytical and Statistical Approaches

On the basis of a previous study [30], we estimated that a sample size of 180 participants would provide 80% power at a two-tailed 5% significance level to detect a difference in HbA_1c_ of 0.4% between the 2 arms with three repeated measures based on a previous study. This estimation assumed a 20% loss to follow-up, a SD change of 1% in HbA_1c_, and an intraclass correlation coefficient of 0.6 within measurements from the same participant.

All enrolled participants who completed the baseline assessment were included in the *intention-to-treat* analysis [31]. Descriptive analyses were conducted for baseline variables, and *t* tests or chi-square tests were performed (as appropriate) to determine any differences in baseline characteristics between the 2 study arms. For indicators related to program use, mean with SD and range were reported for key metrics. A *chat* was included in the analysis of chat duration only if the whole appointment module with *Laura* was completed during the chat (ie, valid values were available in the program data). Durations were truncated when the records showed extreme values (larger than two interquartile ranges above the third quartile of the distribution), as they were likely to represent cases when participants had forgotten to exit the app and duration had been tracked beyond the end of the actual chat.

The main effectiveness analysis followed the intention-to-treat principle [31]. The analysis fitted a linear mixed-effect model for repeated measures over the 3 time points of data collection, with a random intercept for the participant and fixed effects for study arm, time point, and study arm-by-time interaction. Two sets of sensitivity analyses were performed: (1) adjusted model: adjusted baseline covariates that were identified as being imbalanced between the two arms or significantly associated with lost-to-follow-up and (2) per-protocol analysis: intervention participants who had completed ≥7 completed chats with *Laura*. Posthoc subgroup analyses were conducted based on age, sex, baseline HbA_1c_, and NDSS registration duration by adding the subgroup variable and its interaction term with the variable of the interaction between intervention and assessment point as fixed effects to the mixed-effect models used for the main analysis. To further investigate the relationship between program use and effectiveness, mixed-effect models were fitted with a random intercept for the participant and fixed effect for levels of program use (number of chats as 0-6, 7-24, and 25+), assessment time point, and program use-by-time interaction. All analyses were conducted in Stata version 15.1 (Stata Corp) by EG and verified by a statistician (ML) who was blinded to the randomization allocation.

## Results

### Participant Flow and Baseline Characteristics

**Figure 1:**
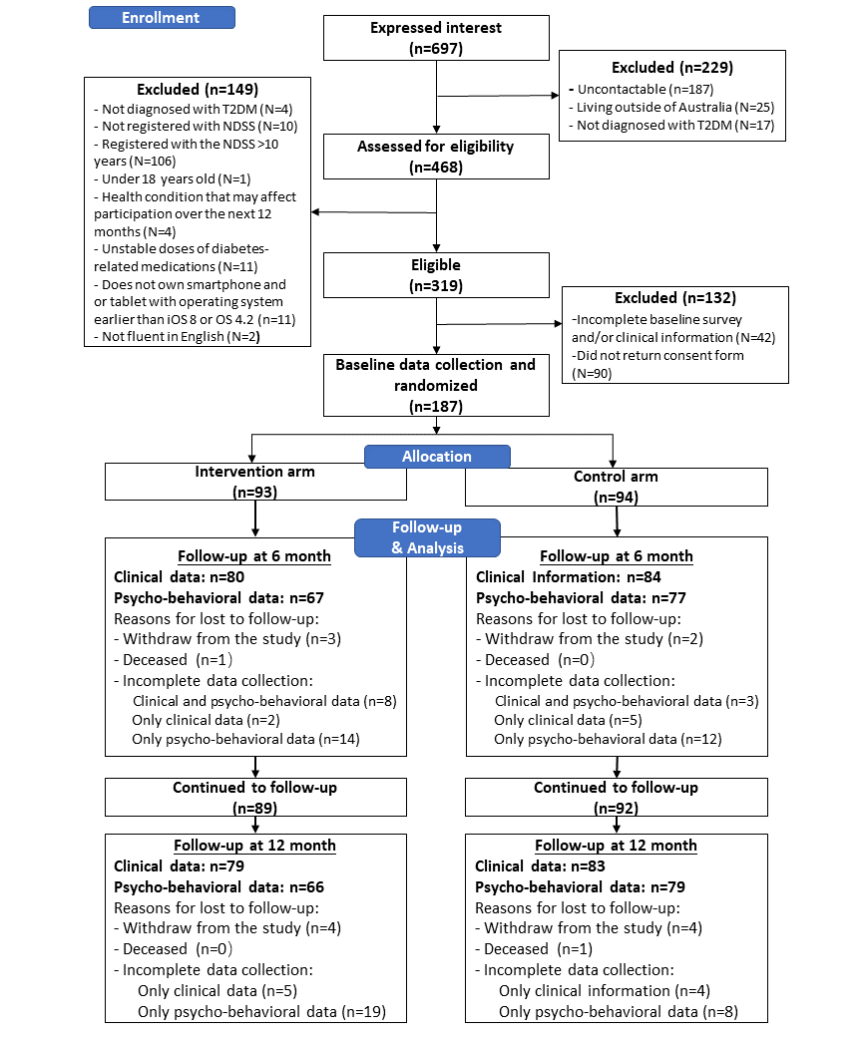
Enrollment, randomization, and follow-up of study participants.

Of the 697 individuals with T2DM who expressed interest in participating in the study, 187 were recruited, including 62 (33%) from Victoria, 21 (16.5%) from New South Wales, and 21 (16.5%) from Queensland (Figure 1). The sample included 78 (41.7%) women, with a mean age of 57 (SD 10) years, 76 (40.7%) had a bachelor’s degree or above, 88 (47.1%) worked full time, and 72 (38.5%) were registered with the NDSS within the 12 months before recruitment (Table 1). The baseline mean HbA_1c_ was 7.3% (56 mmol/mol, SD 1.5%). Individuals who were excluded were more likely to be females, have relatively lower education levels, be unemployed, and use mobile phones less or do not have access to a smartphone (Multimedia Appendix 2).

The intervention and control arms (n=93 and n=94, respectively) were generally comparable, except that those in the intervention arm were slightly younger (*P*=.04) and had lower depression scores (*P*=.004) at baseline. Among the participants, 116 (62.0%) provided complete data for all 3 assessments (baseline, 6, and 12 months). Incomplete assessments were due to active withdrawal, loss to follow-up, incomplete surveys, or refusal to complete a clinical measure with blood tests. Participants without HbA_1c_ measures provided by GPs at 6 months or 12 months were more likely to have lower baseline HRQoL (*P*=.01) and higher anxiety symptom scores (*P*=.03). Participants who had an incomplete survey without a valid AQoL-8D score were younger (*P*=.02) and more likely to be allocated to the intervention arm (*P*=.007).

**Table 1:** Baseline characteristics of study participants

Baseline characteristics			Waitlist (n=94)	Intervention (n=93)	Total (N=187)	*P* value
**Demographic characteristics**						
	Age^a^, (years), mean (SD)		58.4 (10.5)	55.4 (9.7)	56.9 (10.2)	.04
	**Sex, n (%)**					.12
		Male	60 (63.8)	49 (52.7)	109 (58.3)	
		Female	34 (36.2)	44 (47.3)	78 (41.7)	
	**Education, n (%)**					.25
		Secondary high school or lower	29 (30.8)	25 (26.9)	54 (18.8)	
		Technical apprenticeship or diploma	30 (31.9)	27 (29.0)	57 (30.5)	
		Bachelor’s degree	23 (24.5)	17 (18.3)	40 (21.4)	
		Postgraduate degree or higher	12 (12.8)	24 (25.8)	36 (19.3)	
	**Employment, n (%)**					.99
		Full time	43 (45.7)	45 (48.4)	88 (47.1)	
		Part time or casual	16 (17.0)	14 (15.1)	30 (16.0)	
		Retired	21 (22.3)	21 (22.6)	42 (22.5)	
		Unemployed or others	14 (14.9)	13 (14.0)	27 (14.4)	
	English as a secondary language, n (%)		11 (11.7)	6 (6.5)	17 (9.1)	.21
	Aboriginal or Torres Strait Islander origin, n (%)		0 (0.0)	4 (4.3)	4 (2.1)	.08
	General app use: frequent (multiple times per day), n (%)^b^		67 (71.3)	69 (74.2)	136 (72.7)	.38
**Psychosocial characteristics**						
	Health-related quality of life: Assessment of Quality of Life-8 Dimensions score, mean (SD)		0.7 (0.2)	0.7 (0.2)	0.7 (0.2)	.13
	**Depressive symptoms: HADS-D^c^ score, mean (SD)^a^**		4.7 (3.3)	3.3 (3.4)	4.0 (3.4)	.004
		Cases (or likely cases) of depression, n (%)	24 (25.5)	12 (12.9)	36 (19.3)	.03
	**Anxiety symptoms: HADS-A^d^ score, mean (SD)**		5.6 (3.3)	5.4 (3.8)	5.5 (3.5)	.67
		Cases (or likely cases) of anxiety, n (%)	27 (28.7)	28 (30.1)	55 (29.4)	.84
	**Diabetes-specific distress: PAID^e^ score, mean (SD)**		30.5 (19.9)	29.2 (21.4)	29.9 (20.6)	.67
		Severe diabetes distress (PAID score >40), n (%)	31 (33.0)	29 (31.2)	60 (32.1)	.79
**Diabetes-related/health behaviors**						
	**Years registered with NDSS^f^, n (%)^b^**					.51
		≤1 year	34 (38.2)	38 (42.7)	72 (40.5)	
		1-5 years	27 (30.3)	23 (25.8)	50 (28.1)	
		6-10 years	28 (31.5)	28 (31.5)	56 (31.5)	
	**Diabetes medications, n (%)**					
		Diabetes medication(s) prescribed	83 (88.3)	80 (86.0)	163 (87.2)	.64
		Insulin prescribed	16 (17.0)	15 (16.1)	31 (16.6)	.06
		Taking medicines daily as recommended	71 (75.5)	74 (79.6)	145 (77.5)	.33
	Smoking**,** n (%)		7 (7.5)	4 (4.3)	11 (5.9)	.16
	Self-monitoring of blood glucose (>5 days in past 7 days), n (%)^b^		49 (52.1)	53 (57.0)	102 (54.5)	.49
	Daily foot checks**,** n (%)		23 (24.5)	21 (22.6)	44 (23.5)	.87
**Clinical characteristics**						
	Weight (kg), mean (SD)^b^		95.7 (19.0)	97.1 (22.5)	96.4 (20.8)	.65
	Glycated hemoglobin (%), mean (SD)		7.3(1.6)	7.3(1.5)	7.3 (1.5)	.86
	Total cholesterol (mmol/L), mean (SD)^b^		4.5 (1.3)	4.6 (1.4)	4.6 (1.3)	.54
	Systolic blood pressure (mm Hg), mean (SD)^b^		130.4 (13.6)	131.1 (14.6)	130.7 (14.1)	.72
	Diastolic blood pressure (mm Hg), mean (SD)		78.5 (9.3)	78.4 (9.4)	78.5 (9.3)	.94
	Triglyceride (mmol/L), mean (SD)		2.0 (1.3)	1.8 (0.8)	1.9 (1.1)	.26
**Diagnosed comorbidities^b^**						
	High cholesterol, n (%)		59 (62.8)	64 (68.8)	123 (65.8)	.37
	Hypertension, n (%)		52 (55.3)	56 (60.2)	108 (57.8)	.45
	Arthritis (rheumatoid, osteoarthritis, or other), n (%)^b^		34 (36.2)	22 (23.7)	56 (30.0)	.11
	Depression/anxiety/nervous disorder, n (%)		26 (27.7)	26 (28.0)	52 (27.8)	.60
	Heart diseases, n (%)		17 (18.1)	17 (18.3)	34 (18.2)	.60
	Diabetes-related eye complications, n (%)		12 (12.8)	12 (12.9)	24 (12.8)	.60
	Lung diseases, n (%)		11 (11.7)	13 (14.0)	24 (12.8)	.53
	Diabetes-related neuropathy, n (%)		11 (11.7)	11 (11.8)	22 (11.8)	.60
	Stomach, duodenal, or gastro-intestinal ulcer, n (%)		11 (11.7)	10 (10.8)	21 (11.2)	.59
	Cancer, n (%)		6 (6.4)	8 (8.6)	14 (7.5)	.50
	Stroke, n (%)		4 (4.3)	10 (10.8)	14 (7.5)	.14
	Peripheral vascular diseases, n (%)		8 (8.5)	6 (6.5)	14 (7.5)	.53
	Kidney disease, n (%)		5 (5.3)	6 (6.5)	11 (5.9)	.57
**Health care service utilization**						
	Had an appointment with a general practitioner or specialist in the past 12 months, n (%)		93 (98.9)	92 (98.9)	185 (98.9)	.32
	Had an appointment with any other health professional (eg, dietician) in the past 12 months, n (%)		54 (57.4)	63 (67.7)	117 (62.6)	.12
	Admitted to hospital in the past 6 months, n (%)		21 (22.3)	12 (12.9)	33 (17.6)	.10
	Used any other hospital service over the past 6 months that did not involve an admission, n (%)		20 (21.3)	21 (22.6)	41 (21.9)	.80

^a^Significant difference observed between the intervention and control arms.

^b^Some missing values exist: general app use (n=4), years registered with NDSS (n=9), weight (n=8), systolic blood pressure (n=2), diastolic blood pressure (n=2), total cholesterol (n=11), triglyceride (n=13), medication adherence (n=24), self-monitoring of blood glucose (n=6), diagnosed comorbidities (n=1), and health care service utilization (n=1).

^c^HADS-D: Hospital Anxiety and Depression Scale-Depression score.

^d^HADS-A: Hospital Anxiety and Depression Scale-Anxiety score.

^e^PAID: Problem Areas in Diabetes scale.

^f^NDSS: National Diabetes Service Scheme.

### Program Adoption and Use

Table 2 shows the program adoption and use among the 93 participants in the intervention arm. During the 12-month program access, 92 participants (98.9%) completed at least one chat with *Laura*, and 83 participants (89.2%) uploaded their blood glucose levels at least once to the app. These 92 participants completed a total of 1942 chats over 12 months of program access and reached a mean of 243 (SD 212) min of chats per person. In general, the program use, including the number of chats and number of blood glucose uploads, reduced over time of the program access (Multimedia Appendix 2). During the 12-month program access, participants who had at least one chat per month with Laura reduced from 87% in the first month (n=81) to 15% (n=14) in the 12th month, and participants who kept recording their blood glucose in the app dropped from 78% (n=73) to 23% (n=21). In addition, the system generated 297 clinical alerts (related to issues on glucose levels, foot ulcer management, and medication side effects) and 179 technical alerts (eg, glucose uploading failed or user did not interact with the app for 14 days) to the program coordinator. A total of 19 discussion topics were posted on the web-based discussion forum covering a broad range of topics such as stigma and social support, depression, and stress management to facilitate the discussion among participants in the intervention arm.

**Table 2:** Indicators of program adoption and use among participants in the intervention arm (My Diabetes Coach app).

Indicators of program adoption and use			Intervention arm (n=93)
**Uptake of the MDC^a^app, n (%)**			
	Participants who had at least one “appointment” with “Laura” over 12 months		92 (99)
	Participants who had uploaded glucose data into the MDC app		83 (89)
	**Number of completed chats with “Laura” over 12 months, n (%)**		
		0-6	26 (28)
		7-24	37 (40)
		25 or more	30 (32)
	Total number of chats completed over 12 months		1942
	Total number of valid chats completed over 12 months^b^		1641
**Individual-level MDC app usage^c^, mean (SD); range**			
	Number of chats completed per person		21.8 (16.7); 1-65
	Number of valid chats completed per person^b^		18.4 (15.0); 1-53
	Duration of valid chats per person (in minutes), mean (SD); range^d^		
		Total duration of chats	242.7 (212.3); 0-1050
		Mean duration of each valid chat	13.4 (4.8); 3-26.8
	Glucose data uploaded, mean (SD); range		
		Number of glucose level uploads per person	181.8 (192.1); 1-966
**Program delivery,** **total; mean per month (SD)**			
	Clinical alerts (eg, abnormal glucose level)		297; 13.7 (8.8)
	Technical alerts (eg, glucose uploading failed)		179; 8.3 (6.5)
	Posts on the web-based discussion forum		19; 1.1

^a^MDC: My Diabetes Coach.

^b^Invalid chats were defined as chats for which participants exited the app before the coach modules were fully completed with the closing remark.

^c^For individual-level information, the estimation is based on 92 participants who had records of chat with Laura through the app and 83 participants who had uploaded their glucose levels into the app. Mean (SD) and range of number and duration of chats and glucose data uploads were reported.

^d^Only completed chats have been included in the calculation of the total duration of chats. If the users did not exit the app after completing the chats, the duration would be continuously counted. Thus, we truncated the values if the duration of the chats were more than two interquartile ranges above the third quartile of the distribution.

### Program Effectiveness

There was a statistically significant between-arm difference at 12 months in the mean change in HRQoL (AQoL-8D utility value: 0.04, 95% CI 0.00 to 0.07; *P*=.04) but not in HbA_1c_ (−0.04, 95% CI −0.45 to 0.36; *P*=.83; Table 3). The MDC arm had a significant improvement in HRQoL from baseline to 12 months (mean estimated change of AQoL-8D score: 0.04, 95% CI 0.01 to 0.06; *P=*.007)*.* This was maintained from the increase at 6 months (mean estimated change: 0.05, 95% CI 0.01 to 0.08; *P*=.006). HRQoL remained stable for participants in the control arm between baseline and 12 months (mean estimated change: −0.00, 95% CI −0.03 to 0.02; *P*=.92). The adjusted model showed consistent results. The per-protocol analysis yielded a slightly larger effect size, as the 12-month between-arm difference in the AQoL-8D score was 0.06 (95% CI 0.02 to 0.09; *P*=.003). Compared with baseline, the intervention resulted in an increase in the score of the physical health and mental health subscales (Multimedia Appendix 2). There were significant between-arm differences in the mean change of mental health subscore at 6 months as well as the mean change of physical health subscore at 12 months.

**Table 3:** Effectiveness of the intervention on coprimary outcomes.

Coprimary outcomes and analysis models and Arms			Between arm differences at 6 months (95% CI)	*P* value	Between arm differences at 12 months (95% CI)	*P* value	Estimated mean changes between baseline and 6 months (95% CI)^a^	*P* value	Estimated mean changes between baseline and 12 months (95% CI)^a^	*P* value
**Glycated hemoglobin** **(%)**										
	**Primary (Intention-to-treat) model^b^**									
		Intervention	0.06 (−0.35 to 0.47)	.78	−0.04 (−0.45 to 0.36)	.84	−0.20 (−0.49 to 0.09)	.17	−0.33 (−0.62 to −0.04)	.03
		Control	Reference	N/A^c^	Reference	N/A	−0.26 (−0.55 to 0.03)	.08	−0.28 (−0.57 to 0.00)	.05
	**Adjusted model^d^**									
		Intervention	0.06 (−0.35 to 0.46)	.79	−0.04 (−0.44 to 0.37)	.87	−0.20 (−0.49 to 0.09)	.18	−0.32 (−0.61 to −0.03)	.03
		Control	Reference	N/A	Reference	N/A	−0.25 (−0.54 to 0.03)	.09	−0.28 (−0.57 to 0.00)	.05
	**Per-protocol model^e^**									
		Intervention	−0.05 (−0.47 to 0.37)	.81	−0.14 (−0.56 to 0.28)	.52	−0.26 (−0.59 to 0.07)	.12	−0.40 (−0.73 to −0.06)	.02
		Control	Reference	—	Reference	—	−0.21 (−0.47 to 0.05)	.11	−0.26 (−0.51 to −0.00)	.05
**Health Related Quality of Life: Assessment of Quality of Life-8D utility score**										
	**Primary (Intention-to-treat) model^b^**									
		Intervention	0.05 (0.01 to 0.08)	.006	0.04 (0.00 to 0.07)	.039	0.04 (0.01 to 0.07)	.002	0.04 (0.01 to 0.06)	.007
		Control	Reference	N/A	Reference	N/A	−0.01 (−0.03 to 0.02)	.48	0.00 (−0.03 to 0.02）	.92
	**Adjusted model^d^**									
		Intervention	0.05 (0.01 to 0.08)	.005	0.03 (0.00 to 0.07)	.047	0.04 (0.01 to 0.06)	.002	0.03 (0.01 to 0.06)	.009
		Control	Reference	N/A	Reference	N/A	−0.01 (−0.03 to 0.01)	.46	−0.00 (−0.02 to 0.02)	.93
	**Per-protocol model^e^**									
		Intervention	0.06 (0.02 to 0.09)	.002	0.06 (0.02 to 0.09)	.003	0.05 (0.02 to 0.08)	.001	0.05 (0.02 to 0.08)	.001
		Control	Reference	N/A	Reference	N/A	−0.01 (−0.03 to 0.01)	.49	−0.01 (−0.03 to 0.02)	.63

^a^Mean changes in outcomes were estimated based on the linear mixed-effect regression model.

^b^For HbA_1c_, the intraclass correlation coefficient (ICC) for the primary model was 0.551 (95% CI 0.465-0.634). For HRQoL, the ICC for the unadjusted model was 0.847 (95% CI 0.806-0.880). Number of participants with valid data at each time point: n for HbA_1c_ (intervention vs control): 93 vs 94 at baseline, 78 vs 78 at 6 months, and 77 vs 79 at 12 months. Number of participants at each time point for HRQoL (intervention vs control): 93 vs 94 at baseline, 67 vs 77 at 6 months, and 60 vs 78 at 12 months.

^c^N/A: not applicable.

^d^The adjusted model adjusted baseline values of variables that were either imbalanced by intervention allocation by chance (baseline age and depression score) or associated with loss to follow-up (baseline AQoL-8D utility value and HADS Anxiety score).

^e^The per-protocol analysis considered participants who had completed more than 6 chats with Laura as following the study protocol.

Compared with baseline, the mean estimated HbA_1c_ decreased in both arms at 12 months (intervention arm: mean estimated change: −0.33%, 95% CI −0.62 to −0.04; *P*=.03 and control arm: −0.28%, 95% CI −0.57 to 0.00; *P*=.05). The results were consistent in the adjusted model. In the per-protocol model, a larger between-arm difference was observed at 12 months (between-arm difference: −0.14%, 95% CI −0.56 to 0.28; *P*=.52).

There was a dose-response relationship between the number of chats and the change in the HRQoL score (Multimedia Appendix 2). Compared with people who completed chats less than 7 times, those who completed more than 24 chats with *Laura* during program access had a significantly greater improvement in AQoL-8D utility score (between-arm difference: 0.09, 95% CI 0.02 to 0.17; *P*=.02) at 12 months. There was no significant difference in HbA_1c_ (−0.33%, 95% CI −1.11 to 0.44; *P*=.40).

Although this study was not powered for subgroup analyses, the results did show some statistically significant differences favoring the intervention for HbA_1c_. There were greater between-arm differences at 6 and 12 months in the mean change in HbA_1c_ for those with higher baseline HbA_1c_ and those registered on the NDSS within the previous year (Multimedia Appendix 2). There were no significant differences in HRQoL at either 6 or 12 months in the sex and age subgroups. The results for secondary outcomes are presented in Multimedia Appendix 2. A significant between-arm difference (−0.89, 95% CI −1.74 to −0.04; *P*=.04) was observed in the mean change in the HADS anxiety score at 6 months but not at 12 months or for other secondary outcomes reported here.

## Discussion

This study is among the very few randomized controlled trials that have evaluated the adoption, use, and effectiveness of a mobile app–based, interactive, embodied conversational agent to support diabetes self-management within home settings over a 12-month period. Our study adds new evidence to this emerging field by demonstrating the program use in a home setting and the effectiveness of app-based interactive conversational agents in supporting diabetes self-management. The MDC program was feasible and shown to be effective in improving participants’ HRQoL. Although HbA_1c_ levels reduced during the trial, the between-arm difference was not statistically significant at 6 or 12 months.

Despite the growing number of studies using mobile technologies for diabetes management [10,11,15], the effectiveness of introducing apps to people with T2DM to support their self-management at large scale remains uncertain given the generally poor quality of apps [12,13,32], unmet consumer needs [14], and studies lacking robust designs with long-term evaluations [10,15]. The MDC program is highly innovative with its conversational element. The MDC app incorporated interactive voice recognition and an embodied conversational agent, *Laura*, with human-like characteristics who used a very conversational style of speech to provide people with T2DM with personalized coaching and support on a range of essential diabetes self-management activities in their home environment. The process evaluation that received response from 66 out of 93 participants at 6 months showed that more than 80% of them considered *Laura* as a helpful, friendly and competent assistant and 72% described *Laura* as trustworthy [33].

The indicators for program use suggest successful uptake among individuals with T2DM, but maintaining long-term program use still remains a challenge. Interestingly, program exposure (an average of 18 *appointments* with *Laura*) is very similar to the TLC diabetes program previously developed and evaluated by our team in Australia [34]; however, the program is more intensive than a human-delivered program that is widely available in Australia (a maximum of eight face-to-face group sessions per calendar year) [35]. Some gamification elements and human-like characteristics were utilized in the MDC program design, as suggested by previous studies [36]. The recently published and separate evaluation from MDC users demonstrated that these techniques did increase users’ engagement with the program but were insufficient to ensure engagement among all users [37]. Program users have diverse preferences in terms of program interaction, which is likely determined by their technology literacy, personal preference, and their existing self-management style [37]. The decreasing trend in program use over time and the dose-response relationship between the level of app use and its effectiveness suggest that more efforts are still required to improve the maintenance of program use over time.

We observed a modest but statistically significant improvement in HRQoL at 6 months, which was maintained at 12 months. This finding is consistent with some previous research, which indicates a small but statistically significant benefit from mobile app–based interventions on HRQoL [15]. The improvement in AQoL utility score was relatively small in scale (0.04 increase from baseline to 12 months), but previous literature indicates that even a small effect size for HRQoL may bring substantial well-being benefit in the long term [8,27,38]. In addition, the magnitude of HRQoL improvement is associated with the dose of program use among individuals who accessed the program; this demonstrates the importance of increasing program use in future studies. Such HRQoL improvement was contributed by the improvement in both mental health and physical health, as indicated by the subscale analysis of AQoL. The reduced anxiety symptoms, indicated by the significant between-arm difference in anxiety scores at 6 months, further confirmed the impact of the program on mental health. Although the intervention reduced HbA_1c_, it did not achieve a significant reduction compared with previous programs [15,39], and there are several likely reasons for this. First, the baseline HbA_1c_ level was not an inclusion criterion, and 52.4% of participants had an HbA_1c_ in the recommended target range (<7% or 53 mmol/mol) at baseline. However, the significant difference by baseline HbA_1c_ observed in the subgroup analysis suggests that the intervention may have significant benefits if the study had targeted only those individuals with HbA_1c_ above target. Second, similar to some previous digital health trials [40], there was a reduction in HbA_1c_ among participants in the control arm. This could be due to the Hawthorne effect with participants sufficiently motivated to be involved in the trial are likely to seek better self-management through other options when they were not allocated to the active intervention. Third, participants’ lack of maintenance in program use may have led to a relatively low dose of program exposure. The larger between-arm differences observed in the per-protocol analysis and the greater impact among people with higher program exposure indicates that the effect of the MDC program could have been larger with better fidelity to the study protocol.

This study has several important strengths. First, study participants were recruited from across Australia, with broad inclusion criteria, and the program was delivered within participants’ home settings. Thus, the sample is broadly representative of Australians with T2DM, and the study findings are likely to be generalizable and scalable. Second, the study followed participants over 12 months, which is a relatively long term compared with many studies of this kind [15]. Third, we applied intention-to-treat and per-protocol analysis and performed further analysis of the relationship between program use and effectiveness. As a pragmatic behavioral trial, applying both analysis frameworks provides evidence on the effectiveness of the intervention, both for the ideal situation based on the protocol design and for the real-world situation with adaptation among users.

There were also some limitations. First, due to the nature of the intervention, we were not able to blind participants or their GPs (who provided clinical measurements) to the study arm allocation. Without being able to blind participants, self-report bias and Hawthorne effects may exist. Second, we observed a higher rate of completed assessments among participants in the control arm than in the intervention arm, possibly due to their interest in gaining program access or because of higher attrition in the intervention arm. Third, due to the relatively small sample size, the subgroup analyses should be interpreted with caution. Although the main analyses were fully powered, the subgroup analysis was underpowered and multiple testing would have increased the likelihood of false positives. Overall, the sample was not dissimilar from previous trials in this field [15].

### Conclusions

To summarize, this study presents findings concerning the effectiveness, adoption, and use of the MDC program, an app-based interactive embodied conversational agent, in supporting individuals with T2DM. Participants had good adoption of the program and completed a significant amount of chats with *Laura* over 12 months. The trial demonstrated benefits for HRQoL among individuals with T2DM in Australia, which remained apparent at 12 months. The lack of effect for HbA_1c_ is likely due to the relatively low baseline HbA_1c_ level and declining program use over time. Strategies that would increase the program engagement and maintenance in use and improve the effect on HbA_1c_ are required before program scale-up.

## Appendix

Multimedia Appendix 1 Introduction about My Diabetes Coach Program and its five components.

Multimedia Appendix 2 Supplementary tables and figures.

Multimedia Appendix 3 CONSORT-eHEALTH checklist (V1.6.1).

## Abbreviations

AQoL-8D: Assessment of Quality of Life-8 Dimensions
CONSORT: Consolidated Standards of Reporting Trials
GP: general practitioner
HADS: Hospital Anxiety and Depression Scale
HbA_1c_: glycated hemoglobin
HRQoL: health-related quality of life
MDC: My Diabetes Coach
NDSS: National Diabetes Service Scheme
NHMRC: National Health and Medical Research Council
T2DM: type 2 diabetes mellitus
TLC diabetes: Telephone-Linked Care for Diabetes
