# Supplementary material for: My Diabetes Coach, a Mobile App–Based Interactive Conversational Agent to Support Type 2 Diabetes Self-Management: Randomized Effectiveness-Implementation Trial
Source: J Med Internet Res. 2020 Nov 5;22(11):e20322. doi: 10.2196/20322 (PMC7677021; doi:10.2196/20322)
Supplement: Multimedia Appendix 1 [file jmir_v22i11e20322_app1.docx]

##### **Supplementary Appendix 1: Introduction about My Diabetes Coach Program and its five components**

**Introduction about My Diabetes Coach**

The My Diabetes Coach program comprises five complementary components, including a smart device application (app), a printed user guide, a program website with a user discussion board, an optional blood glucose (BG) meter with Bluetooth capability (Accu-Chek Aviva Connect®, Roche Diabetes Care), and structured interactions with a program coordinator.


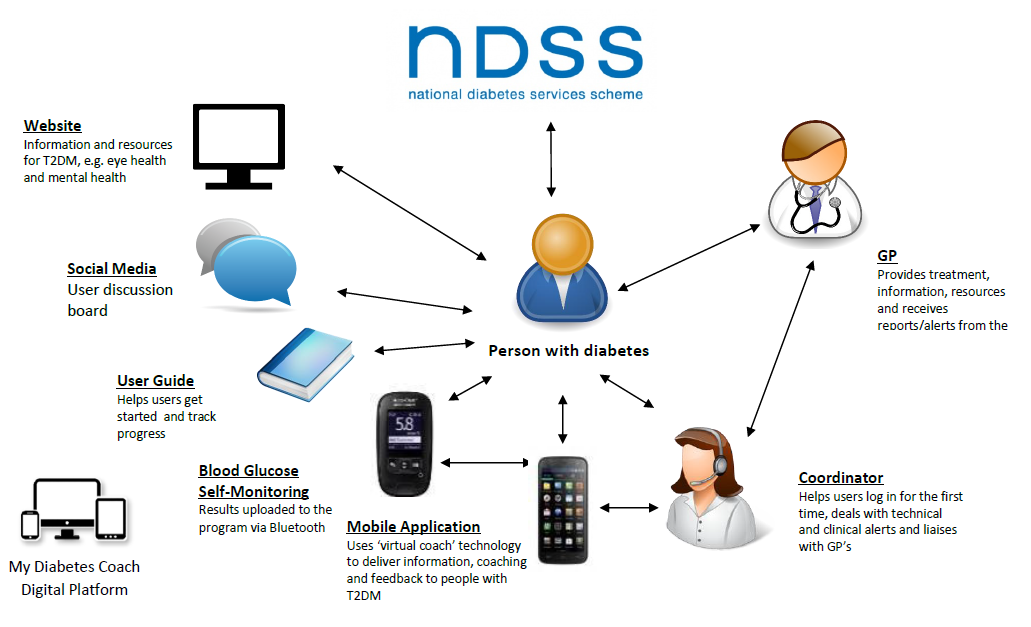


Overview of the My Diabetes Coach program

- **MDC Application**

The MDC app was adapted from the Australian Telephone-Linked Care (TLC) Diabetes program ^[[1]](#footnote-1)^and was designed to deliver personalized support, monitoring, and motivational coaching via an interactive embodied conversational agent, ‘Laura’. The coaching was delivered through weekly ‘appointments’ with ‘Laura’ to emulate weekly appointments with a health coach. ‘Laura’ is an embodied character with a human-like range of facial and gesture animations who mimics human conversation using interactive voice recognition and a database of pre-scripted conversations that uses algorithms and ‘smart’ script logic to personalize the conversations for the user. ‘Laura’ delivers spoken dialogue with lip synchronization and responds in real-time to voice and touch commands. The software supports the detection and capture of speech audio and translation of speech into text based on current dialogue context and speech-driven responses to notifications and interactions.

Screenshot of the app on Laura


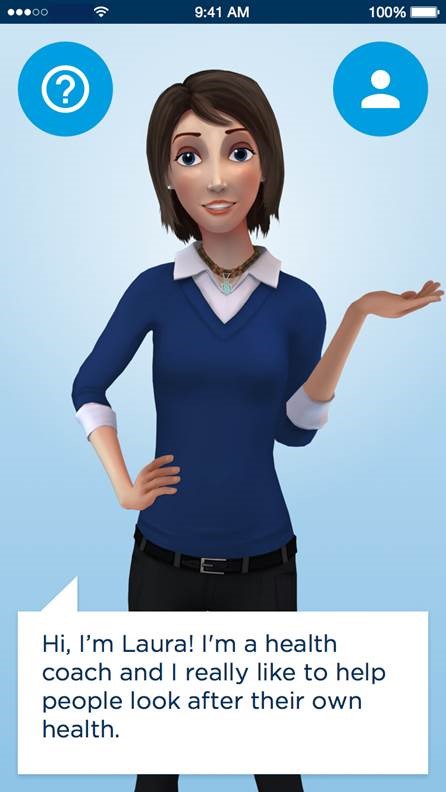


By listening to Laura speak (or by reading the text on the screen) and speaking or touching the screen to reply, MDC app users participate in weekly ‘appointments’ with ‘Laura’ on modules related to diabetes management. The topics covered in the modules were determined by a team of Australian clinical experts, with the aim of covering key elements of diabetes self-care, including blood glucose monitoring, healthy eating, physical activity, medication taking and foot care. The conversational scripts and the algorithms guiding each individual’s progress were designed by applying behavior change theories and techniques, including the Transtheoretical Model ^[[2]](#footnote-2)^, Social Cognitive Theory ^[[3]](#footnote-3)^, gamification^[[4]](#footnote-4)^ and concepts derived from chronic disease self-management^[[5]](#footnote-5)^. Health professionals (with expertise in diabetes management) and people with diabetes were consulted extensively to co-develop and refine the scripts and to co-design the other program components. The user experience is personalized and tailored based on the clinical targets and glucose monitoring frequency recommendations provided by users’ GPs. Each module contains 4 to 12 appointments, depending on users’ responses during the interactions, thereby enabling a high degree of program personalization. Users could choose a particular module by themselves but were required to complete the module over a series of appointments before moving to a new one.

- **An optional blood glucose meter with Bluetooth capability**

The app also enabled blood glucose monitoring. Users could manually upload their glucose measures in the system, or they were provided with an Accu-Chek Aviva Connect ® blood glucose meter with Bluetooth capabilities (Roche Diabetes Care) with no cost, at the beginning of the study. This type of blood glucose meter with Bluetooth capabilities enables the automated upload of glucose data into the app.

- **MDC User Guide**

The user guide complemented use of the app and contained information to help participants access and familiarize themselves with the MDC program. The guide included an introduction to the initial set-up of the app, frequently asked questions, an explanation of the usefulness of goal-setting and problem-solving strategies, tracking sheets for participants to record their progress throughout the program, and sources of more information on managing type 2 diabetes.


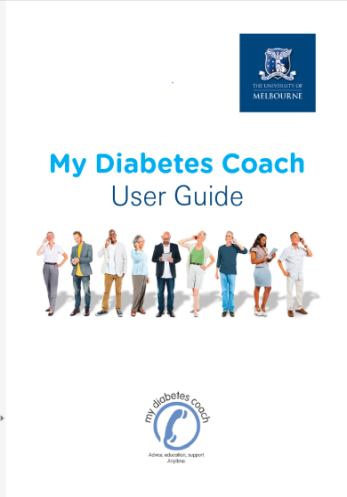

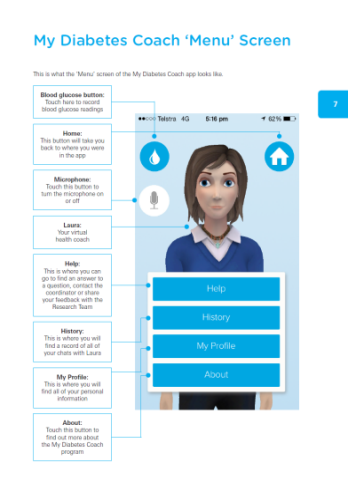

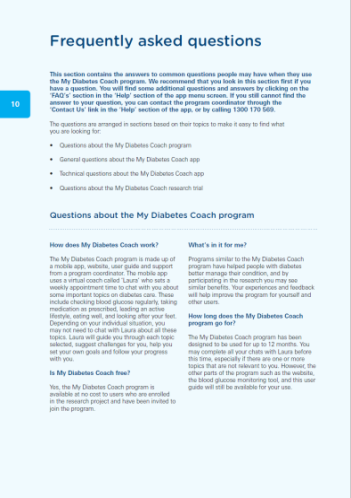


Screenshots of the MDC user guide

- **Website and discussion board**

The MDC website consisted of a publicly accessible project website with information about the program to attract participants and a program website that was only accessible to MDC program users. The program website contained links to recommended resources on diabetes self-care, contact details for diabetes partner organizations, an electronic copy of the user guide, and an embedded online discussion board. The program coordinator posted discussion topics related to diabetes self-management every fortnight and users could share their experiences with other users under the posted topics.


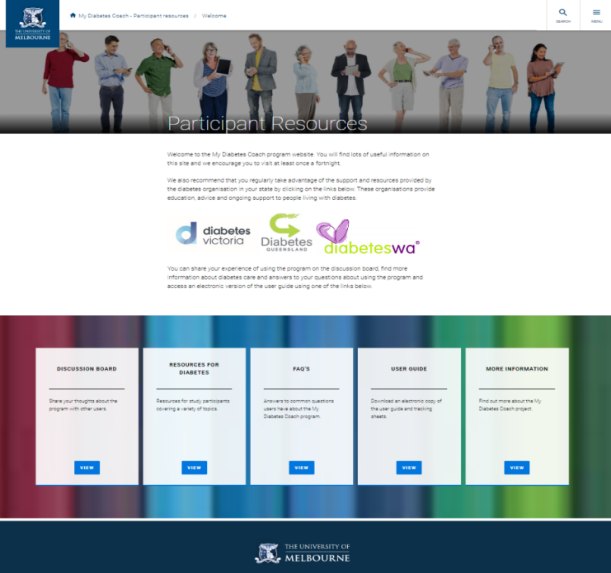

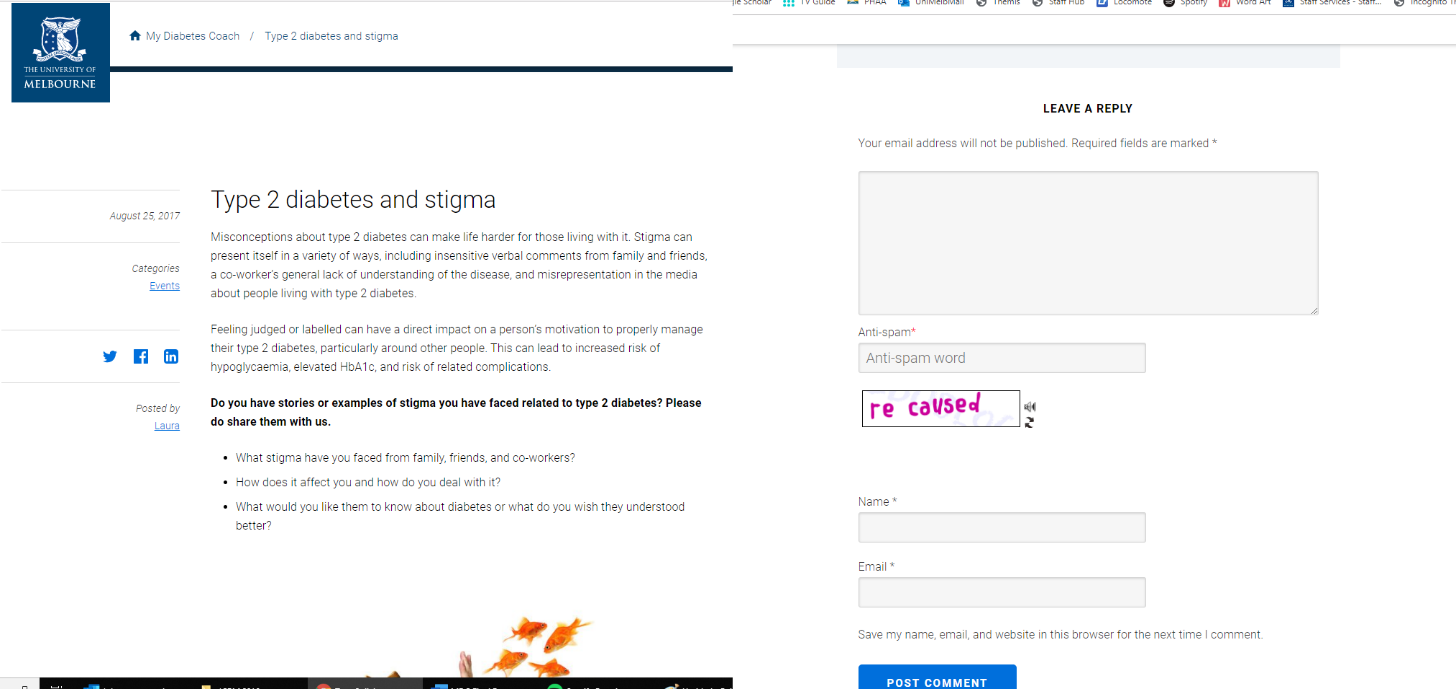


Screenshots of the MDC program website (left) and post for discussion boards (right).

- **MDC program coordinator**

The program coordinator provided technical support to participants. The program coordinator called participants after 1, 4, 8, 12, and 24 weeks during program use to answer any questions, troubleshoot technical issues and help them make the most out of the program. The program coordinator was supported by a web-based user management portal, which allowed them to set up user profiles, trigger clinical alerts for certain cases of very high or low BG level or technical alerts (e.g. failed BG uploading) derived from the system in real-time and review the summary of program usage. The program coordinator also played a key role in communicating with program users and GPs on notifications and alerts to ensure the successful delivery of the program. The program coordinator called participants after important clinical alerts or technical alerts. Coordinator’s responses to alerts were standardized based on a rigorous protocol to ensure the safety of participants and technical alerts were dealt by contacting Clevertar via a Jira system. Most issues were resolved within a day.

**Program Delivery**

A welcome package was posted to participants allocated to the intervention arm containing a welcome letter informing them of randomization allocation, an Accu-Chek Aviva Connect^®^ BG meter enabling BG data upload (if they had chosen to receive one), and the MDC User Guide. The program coordinator sent an email to participants with instructions about first-time log-in and followed up with participants with a phone call within 48 hours of their first log in to assist with any technical difficulties.

Participants were encouraged to use the MDC app, the MDC website and the user guide regularly. Participants were encouraged to complete one appointment per week with ‘Laura’ through the app. In addition to the MDC app, participants also received the MDC quarterly newsletter containing study updates and useful tips, access to other resources on the MDC website and were encouraged to join the discussion on topics related to the diabetes self-management posted by the program coordinator.

##### **Supplementary Table 1. Data collection by type and measurement**

| Data collection approach | Variable | Measurement |
| --- | --- | --- |
| Psycho-behavioral data via self-report (online questionnaire completion) | Health-related Quality of Life | AQoL-8D |
|  | Anxiety and depressive symptoms | Hospital Anxiety and Depression Scale (HADS) |
|  | Diabetes-specific distress | Problem Areas in Diabetes (PAID) scale |
|  | Perceived support | ENRICHD Social Support Instrument (ESSI) |
|  | Self-efficacy in managing diet | Nutrition Self-efficacy scale |
|  | Physical activity | The Active Australia Survey |
|  | Diabetes self-care activities (footcare, blood glucose testing) | Diabetes Self-care Activities Measure |
|  | Medication use and compliance | Self-report (study-specific questions) |
|  | Health service utilization | Self-report (study-specific questions) |
|  | Smoking status | Self-report (study-specific questions) |
| Clinical data (collected by GPs and faxed to the research team) | HbA1c (% and/or mmol/mol), Total cholesterol (mmol/L), HDL, LDL, triglyceride, Creatinine, eGFR | Pathology testing |
|  | Blood pressure (systolic and diastolic) | Clinical measures |
|  | Body weight | Clinical measures |

##### **Supplementary** **Table 2. Number of completed chats by months of program access and proportion of participants with at least one chat with Laura (n=93)**

| Months since program access | Mean (SD) | Range | Participants with at least one chat with Laura  n (%) |
| --- | --- | --- | --- |
| 1 | 3.4 (1.8) | 0-7 | 81 (87.1%) |
| 2 | 2.6 (1.6) | 0-5 | 78 (83.9%) |
| 3 | 2.0 (1.8) | 0-6 | 63 (67.7%) |
| 4 | 1.7 (1.8) | 0-5 | 52 (55.9%) |
| 5 | 1.5 (1.8) | 0-6 | 45 (48.4%) |
| 6 | 1.2 (1.7) | 0-5 | 36 (38.7%) |
| 7 | 1.3 (1.8) | 0-5 | 38 (40.9%) |
| 8 | 1.1 (1.7) | 0-5 | 36 (38.7%) |
| 9 | 0.9 (1.6) | 0-5 | 27 (29.0%) |
| 10 | 0.8 (1.5) | 0-5 | 24 (25.8%) |
| 11 | 0.7 (1.5) | 0-5 | 19 (20.4%) |
| 12 | 0.5 (1.2) | 0-4 | 14 (15.1%) |

##### **Supplementary Table 3. Subgroup analysis on the between-arm differences for co-primary outcomes**

| **Primary outcomes** | **Subgroups** | **Between-arm difference of mean changes at 6 months***  **(95% CI)** | **P-value** | **P for interaction** | **Between arm difference of mean changes at 12 months ***  **(95% CI)** | **P-value** | **P for interaction** |
| --- | --- | --- | --- | --- | --- | --- | --- |
| **HbA1c (%)** | | | | | | | |
| **Main model** |  | 0.06 (-0.35, 0.47) | 0.780 |  | -0.04 (0.45, 0.36) | 0.834 |  |
| **Sex** |  |  |  | 0.125 |  |  | 0.916 |
|  | **Male** | -0.25 (-0.81, 0.31) | 0.383 |  | -0.09 (-0.65, 0.47) | 0.746 |  |
|  | **Female** | 0.40 (-0.20, 1.00) | 0.190 |  | 0.024 (-0.57, 0.62) | 0.937 |  |
| **Age** |  |  |  | 0.556 |  |  | 0.701 |
|  | **<60 years** | 0.32 (-0.32, 0.96) | 0.324 |  | 0.10 (-0.54, 0.74) | 0.754 |  |
|  | **>60 years** | -0.25 (-0.65, 0.15) | 0.221 |  | -0.21 (-0.61, 0.18) | 0.291 |  |
| **HbA1c at baseline** | |  |  | <0.001 |  |  | <0.001 |
|  | **<7%** | 0.23 (-0.13, 0.58) | 0.216 |  | 0.06 (-0.30, 0.42) | 0.740 |  |
|  | **>7%** | -0.15 (-0.85, 0.55) | 0.668 |  | -0.19 (-0.88, 0.51) | 0.598 |  |
| **NDSS registration duration** | |  |  | 0.029 |  |  | 0.227 |
|  | **<1 year** | -0.13 (-0.90, 0.64) | 0.746 |  | 0.03 (-0.73, 0.79) | 0.944 |  |
|  | **>1 year** | 0.17 (-0.30, 0.63) | 0.474 |  | -0.10 (-0.57, 0.37) | 0.675 |  |
| **Health-related quality of life: AQoL-8D utility score** | | | | | | | |
| **Main model** |  | 0.05 (0.01, 0.08) | 0.006 |  | 0.05 (0.01, 0.08) | 0.006 |  |
| **Sex** |  |  |  | 0.994 |  |  | 0.928 |
|  | **Male** | 0.03 (-0.02, 0.08) | 0.230 |  | 0.03 (-0.02, 0.08) | 0.300 |  |
|  | **Female** | 0.08 (0.03, 0.14) | 0.003 |  | 0.06 (0.01, 0.11) | 0.032 |  |
| **Age** |  |  |  | 0.122 |  |  | 0.309 |
|  | **<60 years** | 0.07 (0.03, 0.12) | 0.001 |  | 0.05 (0.00, 0.09) | 0.047 |  |
|  | **>60 years** | 0.01 (-0.04, 0.07) | 0.635 |  | 0.02 (-0.03, 0.08) | 0.435 |  |
| **HbA1c at baseline** | |  |  | 0.511 |  |  | 0.362 |
|  | **<7%** | 0.06 (0.02, 0.10) | 0.009 |  | 0.04 (-0.01, 0.08) | 0.096 |  |
|  | **>7%** | 0.04 (-0.02, 0.10) | 0.156 |  | 0.04 (-0.02, 0.10) | 0.172 |  |
| **NDSS registration duration** | |  |  | 0.451 |  |  | 0.212 |
|  | **<1 year** | 0.04 (0.02, 0.11) | 0.173 |  | 0.04 (-0.02, 0.11) | 0.204 |  |
|  | **>1 year** | 0.05 (0.01, 0.09) | 0.012 |  | -0.03 (-0.01, 0.07) | 0.127 |  |

All models are unadjusted. NDSS: National Diabetes Service Scheme

*Coefficient of arm-by-time interaction with waitlist as reference group from mixed-effect model based on subgroup analysis by covariate

†*p*-value of interaction term on subgroup variable and arm-by-time interaction term in mixed-effect model with waitlist as reference group

##### **Supplementary Table 4. Between-arm differences in mean change of secondary outcomes at 6 months and 12 months**

| **Outcomes** | **Arms** | **Mean (SD)** | | | **Between-arm differences in mean change** | | | |
| --- | --- | --- | --- | --- | --- | --- | --- | --- |
|  |  | **Baseline** | **6 months** | **12 months** | **At 6 months**  **(95% CI)** | **P-value** | **At 12 months (95% CI)** | **P-value** |
| Depressive symptoms: HADS-D score | Intervention | 3.26 (3.36) | 2.62 (3.27) | 2.58 (3.01) | -0.72 (-1.57, 0.13) | 0.098 | -0.54 (-1.42, 0.35) | 0.236 |
|  | Waitlist | 4. 69 (3.33) | 5.03 (4.39) | 4.55 (4.17) | Reference | - | Reference | - |
| Anxiety symptoms: HADS-A score | Intervention | 5.38 (3.75) | 4.47 (3.63) | 4.55 (3.58) | -0.89 (-1.74, -0.04) | 0.041 | -0.71 (-1.60, 0.18) | 0.118 |
|  | Waitlist | 5.60 (3.30) | 5.75 (3.41) | 5.52 (3.40) | Reference | - | Reference | - |
| Diabetes-specific distress: PAID score | Intervention | 29.23 (21.43) | 19.07 (18.95) | 19.92 (19.55) | -3.79 (-8.74, 1.16) | 0.133 | -2.19 (-7.20, 2.81) | 0.390 |
|  | Waitlist | 30.5 (19.9) | 25.0 (19.02) | 23.97 (20.81) | Reference | - | Reference | - |
| Body weight:  kg | Intervention | 97.1 (22.5) | 94.5 (23.6) | 95.5 (21.9) | -0.19 (-2.51, 2.13) | 0.871 | -0.21 (-2.63, 2.21) | 0.865 |
|  | Waitlist | 94.7 (19.7) | 95.3 (21.5) | 97.1 (22.0) | Reference | - | Reference | - |

All models are unadjusted. HADS-A: Hospital Anxiety and Depression Scale – Anxiety score; HADS-D: Hospital Anxiety and Depression Scale – Depression score; PAID: Problem Areas in Diabetes scale

##### **Supplement Figure 1. Mean change in HbA1c levels and health related quality of life by MDC program usage**


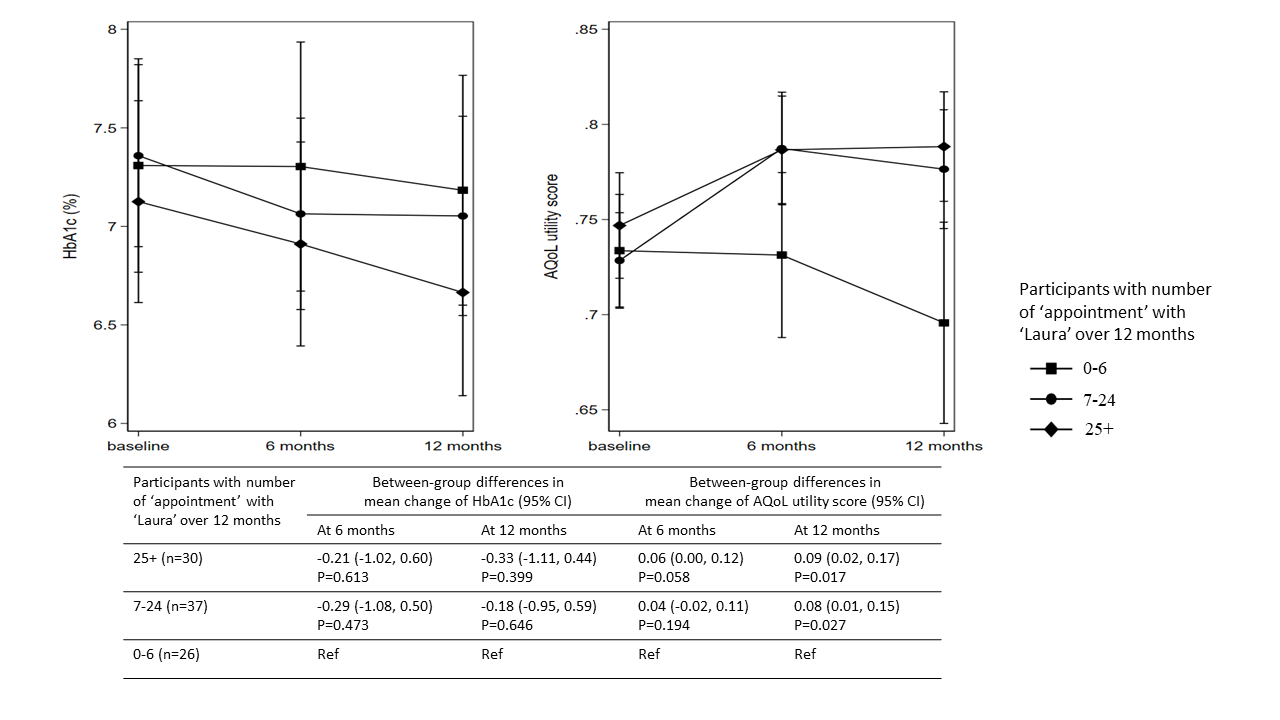


Note: Figures and the table below showed the results from adjusted model where baseline values of variables that are either imbalanced by intervention allocation by chance (baseline age and depression score) or associated with the loss to follow-up (baseline AQoL-8D utility score and HADS Anxiety score).

AQoL: Assessment of Quality of Life

1. Williams ED, Bird D, Forbes AW, Russell A, Ash S, Friedman R, Scuffham PA, Oldenburg B: Randomised controlled trial of an automated, interactive telephone intervention (TLC Diabetes) to improve type 2 diabetes management: baseline findings and six-month outcomes. BMC public health 2012;12:602 [↑](#footnote-ref-1)
2. Prochaska JO, Redding CA, Evers KE: The transtheoretical model and stages of change. Health behavior: Theory, research, and practice 2015:125-148 [↑](#footnote-ref-2)
3. Crosby R, Salazar L, DiCelemente R: Social cognitive theory applied to health behavior. Health Behavior Theory for Public Health: Principles, Foundations, and Applications, edited by Gartside M, Burlington, MA, Jones & Bartlett Learning 2013:163-186 [↑](#footnote-ref-3)
4. Cugelman B: Gamification: what it is and why it matters to digital health behavior change developers. JMIR serious games 2013;1:e3 [↑](#footnote-ref-4)
5. Grady PA, Gough LL: Self-management: a comprehensive approach to management of chronic conditions. Am J Public Health 2014;104:e25-e31 [↑](#footnote-ref-5)
