# Supplementary material for: My Diabetes Coach, a Mobile App–Based Interactive Conversational Agent to Support Type 2 Diabetes Self-Management: Randomized Effectiveness-Implementation Trial
Source: J Med Internet Res. 2020 Nov 5;22(11):e20322. doi: 10.2196/20322 (PMC7677021; doi:10.2196/20322)
Supplement: Multimedia Appendix 2 [file jmir_v22i11e20322_app2.docx]

##### **Supplementary Table 1. Data collection by type and measurement**

| Data collection approach | Variable | Measurement |
| --- | --- | --- |
| Psycho-behavioral data via self-report (online questionnaire completion) | Health-related Quality of Life | AQoL-8D |
|  | Anxiety and depressive symptoms | Hospital Anxiety and Depression Scale (HADS) |
|  | Diabetes-specific distress | Problem Areas in Diabetes (PAID) scale |
|  | Perceived support | ENRICHD Social Support Instrument (ESSI) |
|  | Self-efficacy in managing diet | Nutrition Self-efficacy scale |
|  | Physical activity | The Active Australia Survey |
|  | Diabetes self-care activities (footcare, blood glucose testing) | Diabetes Self-care Activities Measure |
|  | Medication use and compliance | Self-report (study-specific questions) |
|  | Health service utilization | Self-report (study-specific questions) |
|  | Smoking status | Self-report (study-specific questions) |
| Clinical data (collected by GPs and faxed to the research team) | HbA1c (% and/or mmol/mol), Total cholesterol (mmol/L), HDL, LDL, triglyceride, Creatinine, eGFR | Pathology testing |
|  | Blood pressure (systolic and diastolic) | Clinical measures |
|  | Body weight | Clinical measures |

##### **Supplementary** **Table 2. Baseline socio-demographic characteristics among participants included and excluded during the screening stage**

| **Baseline characteristics** | **Enrolled**  **(N = 187)** | **Excluded**  **(N=281)** | **P-value*** |
| --- | --- | --- | --- |
| Age at baseline | 56.9 (10.2) | 57.6 (11.7) | 0.884 |
| Sex |  |  | 0.029 |
| Male | 109 (58.3%) | 131 (46.6%) |  |
| Female | 78 (41.7%) | 148 (52.7%) |  |
| Missing | - | 2 (0.71%) |  |
| Education |  |  | 0.095 |
| Secondary high school or lower | 54 (18.8%) | 55 (19.5%) |  |
| Technical apprenticeship / diploma | 57 (30.5%) | 44 (15.7%) |  |
| Bachelor’s degree | 40 (21.4%) | 21 (7.5%) |  |
| Post-graduate degree or higher | 36 (19.3%) | 18 (6.4%) |  |
| Missing | - | 143 (50.9%) |  |
| Employment |  |  | 0.015 |
| Full-time | 88 (47.1%) | 45 (16.0%) |  |
| Part-time or casual | 30 (16.0%) | 25 (8.9%) |  |
| Retired | 42 (22.5%) | 31 (11.0%) |  |
| Unemployed or others | 27 (14.4%) | 37 (13.2%) |  |
| Missing | - | 143 (50.9%) |  |
| English as a secondary language |  |  | 0.919 |
| Yes | 17 (9.1%) | 13 (4.6%) |  |
| No | 170 (90.9%) | 125 (44.5%) |  |
| Missing | - | 143 (50.9%) |  |
| Aboriginal or Torres Strait Islander origin | |  | 0.425 |
| Yes | 4 (2.1%) | 5 (1.8%) |  |
| No | 182 (97.3%) | 133 (47.3%) |  |
| Missing | 1 (0.53%) | 143 (50.9%) |  |
| General App use |  |  | 0.015 |
| Frequent (multiple times per day) | 136 (72.7%) | 159 (56.6%) |  |
| Less frequent or no access | 50 (26.7%) | 97 (34.5%) |  |
| Missing | 1 (0.53%) | 25 (8.9%) |  |

* P-values were generated by performing the t-test or chi-square tests excluding the missing values.

##### **
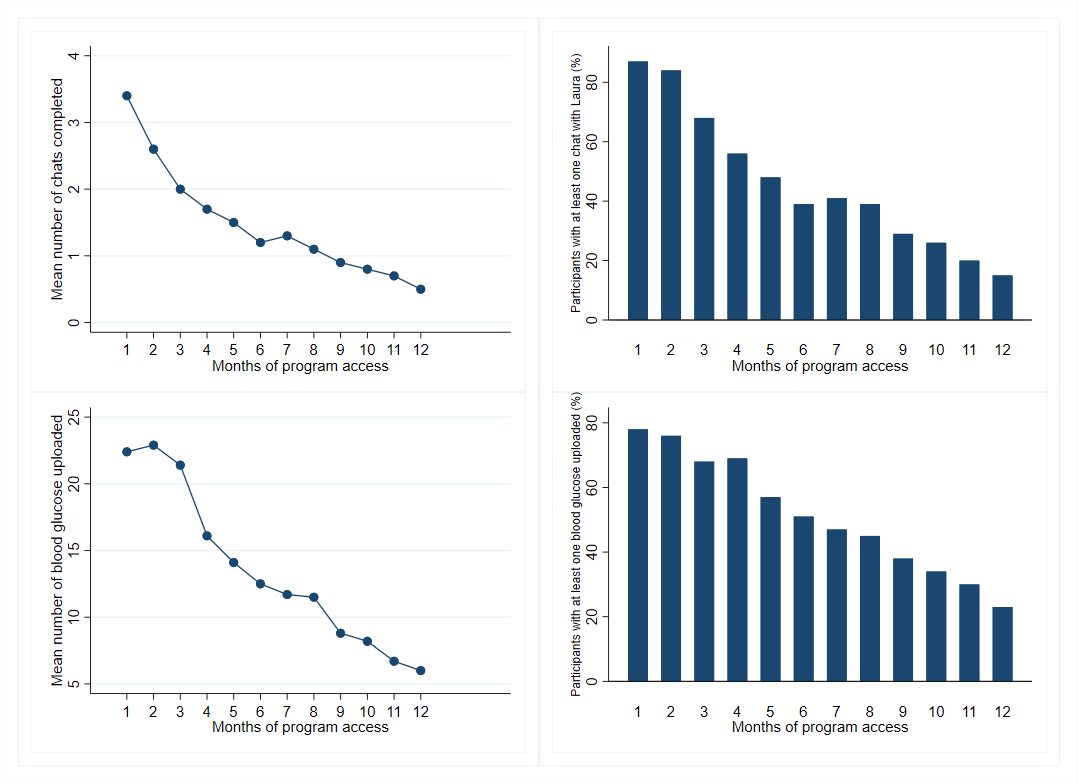
Supplementary Figure 1. Program adoption and use over the 12 months of program access**


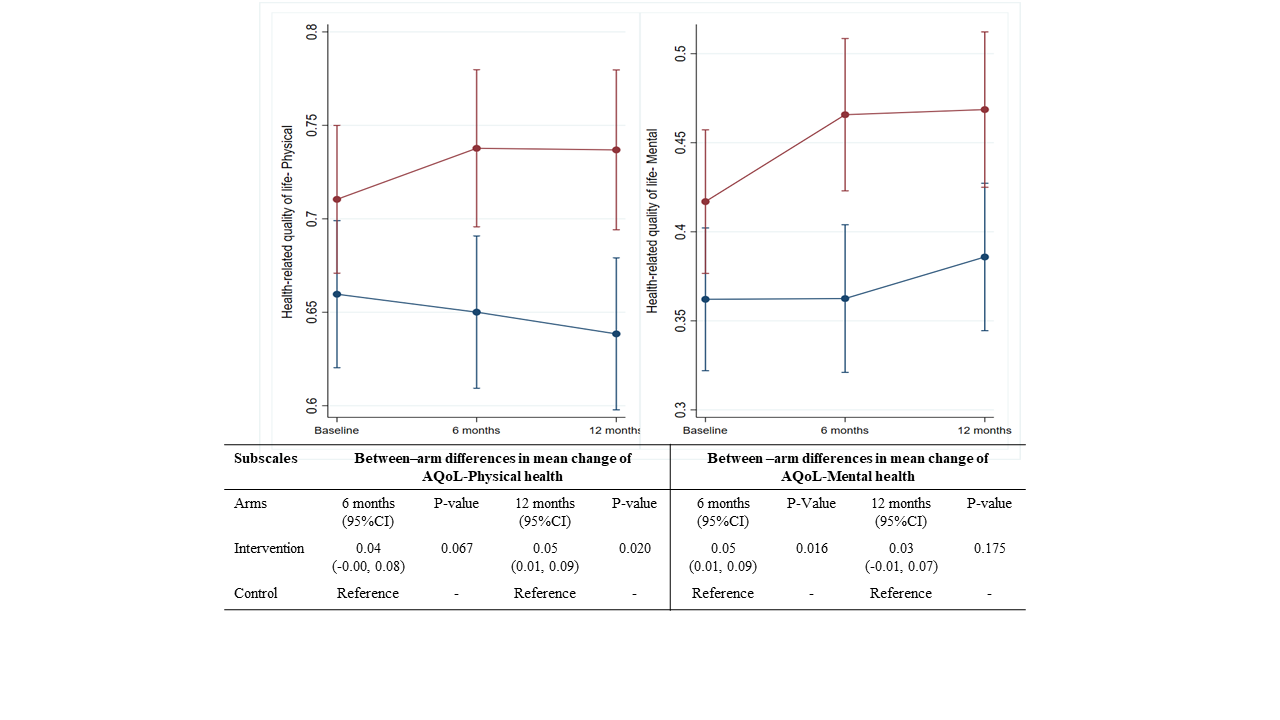


##### **Supplement Figure 2. Effectiveness of the intervention on the subscales of Health-related quality of life**

##### **Supplementary Table 3. Subgroup analysis of between-arm differences for co-primary outcomes**

| **Primary outcomes** | **Subgroups** | **Between-arm difference of mean changes at 6 months***  **(95% CI)** | **P-value** | **P for interaction** | **Between arm difference of mean changes at 12 months ***  **(95% CI)** | **P-value** | **P for interaction** |
| --- | --- | --- | --- | --- | --- | --- | --- |
| **HbA1c (%)** | | | | | | | |
| **Main model** |  | 0.06 (-0.35, 0.47) | 0.780 |  | -0.04 (0.45, 0.36) | 0.834 |  |
| **Sex** |  |  |  | 0.125 |  |  | 0.916 |
|  | **Male** | -0.25 (-0.81, 0.31) | 0.383 |  | -0.09 (-0.65, 0.47) | 0.746 |  |
|  | **Female** | 0.40 (-0.20, 1.00) | 0.190 |  | 0.024 (-0.57, 0.62) | 0.937 |  |
| **Age** |  |  |  | 0.556 |  |  | 0.701 |
|  | **<60 years** | 0.32 (-0.32, 0.96) | 0.324 |  | 0.10 (-0.54, 0.74) | 0.754 |  |
|  | **>60 years** | -0.25 (-0.65, 0.15) | 0.221 |  | -0.21 (-0.61, 0.18) | 0.291 |  |
| **HbA1c at baseline** | |  |  | <0.001 |  |  | <0.001 |
|  | **<7%** | 0.23 (-0.13, 0.58) | 0.216 |  | 0.06 (-0.30, 0.42) | 0.740 |  |
|  | **>7%** | -0.15 (-0.85, 0.55) | 0.668 |  | -0.19 (-0.88, 0.51) | 0.598 |  |
| **NDSS registration duration** | |  |  | 0.029 |  |  | 0.227 |
|  | **<1 year** | -0.13 (-0.90, 0.64) | 0.746 |  | 0.03 (-0.73, 0.79) | 0.944 |  |
|  | **>1 year** | 0.17 (-0.30, 0.63) | 0.474 |  | -0.10 (-0.57, 0.37) | 0.675 |  |
| **Health-related quality of life: AQoL-8D utility score** | | | | | | | |
| **Main model** |  | 0.05 (0.01, 0.08) | 0.006 |  | 0.05 (0.01, 0.08) | 0.006 |  |
| **Sex** |  |  |  | 0.994 |  |  | 0.928 |
|  | **Male** | 0.03 (-0.02, 0.08) | 0.230 |  | 0.03 (-0.02, 0.08) | 0.300 |  |
|  | **Female** | 0.08 (0.03, 0.14) | 0.003 |  | 0.06 (0.01, 0.11) | 0.032 |  |
| **Age** |  |  |  | 0.122 |  |  | 0.309 |
|  | **<60 years** | 0.07 (0.03, 0.12) | 0.001 |  | 0.05 (0.00, 0.09) | 0.047 |  |
|  | **>60 years** | 0.01 (-0.04, 0.07) | 0.635 |  | 0.02 (-0.03, 0.08) | 0.435 |  |
| **HbA1c at baseline** | |  |  | 0.511 |  |  | 0.362 |
|  | **<7%** | 0.06 (0.02, 0.10) | 0.009 |  | 0.04 (-0.01, 0.08) | 0.096 |  |
|  | **>7%** | 0.04 (-0.02, 0.10) | 0.156 |  | 0.04 (-0.02, 0.10) | 0.172 |  |
| **NDSS registration duration** | |  |  | 0.451 |  |  | 0.212 |
|  | **<1 year** | 0.04 (0.02, 0.11) | 0.173 |  | 0.04 (-0.02, 0.11) | 0.204 |  |
|  | **>1 year** | 0.05 (0.01, 0.09) | 0.012 |  | -0.03 (-0.01, 0.07) | 0.127 |  |

All models are unadjusted. NDSS: National Diabetes Service Scheme

*Coefficient of arm-by-time interaction with waitlist as reference group from mixed-effect model based on subgroup analysis by covariate

†*p*-value of interaction term on subgroup variable and arm-by-time interaction term in mixed-effect model with waitlist as reference group

##### **Supplementary Table 4. Between-arm differences in mean change of secondary outcomes at 6 months and 12 months**

| **Outcomes** | **Arms** | **Mean (SD)** | | | **Between-arm differences in mean change** | | | |
| --- | --- | --- | --- | --- | --- | --- | --- | --- |
|  |  | **Baseline** | **6 months** | **12 months** | **At 6 months**  **(95% CI)** | **P-value** | **At 12 months (95% CI)** | **P-value** |
| Depressive symptoms: HADS-D score | Intervention | 3.26 (3.36) | 2.62 (3.27) | 2.58 (3.01) | -0.72 (-1.57, 0.13) | 0.098 | -0.54 (-1.42, 0.35) | 0.236 |
|  | Waitlist | 4. 69 (3.33) | 5.03 (4.39) | 4.55 (4.17) | Reference | - | Reference | - |
| Anxiety symptoms: HADS-A score | Intervention | 5.38 (3.75) | 4.47 (3.63) | 4.55 (3.58) | -0.89 (-1.74, -0.04) | 0.041 | -0.71 (-1.60, 0.18) | 0.118 |
|  | Waitlist | 5.60 (3.30) | 5.75 (3.41) | 5.52 (3.40) | Reference | - | Reference | - |
| Diabetes-specific distress: PAID score | Intervention | 29.23 (21.43) | 19.07 (18.95) | 19.92 (19.55) | -3.79 (-8.74, 1.16) | 0.133 | -2.19 (-7.20, 2.81) | 0.390 |
|  | Waitlist | 30.5 (19.9) | 25.0 (19.02) | 23.97 (20.81) | Reference | - | Reference | - |
| Body weight:  kg | Intervention | 97.1 (22.5) | 94.5 (23.6) | 95.5 (21.9) | -0.19 (-2.51, 2.13) | 0.871 | -0.21 (-2.63, 2.21) | 0.865 |
|  | Waitlist | 94.7 (19.7) | 95.3 (21.5) | 97.1 (22.0) | Reference | - | Reference | - |

All models are unadjusted. HADS-A: Hospital Anxiety and Depression Scale – Anxiety score; HADS-D: Hospital Anxiety and Depression Scale – Depression score; PAID: Problem Areas in Diabetes scale

##### **Supplementary Figure 3. Mean change in glycated hemoglobin levels and health related quality of life by My Diabetes Coach program usage**


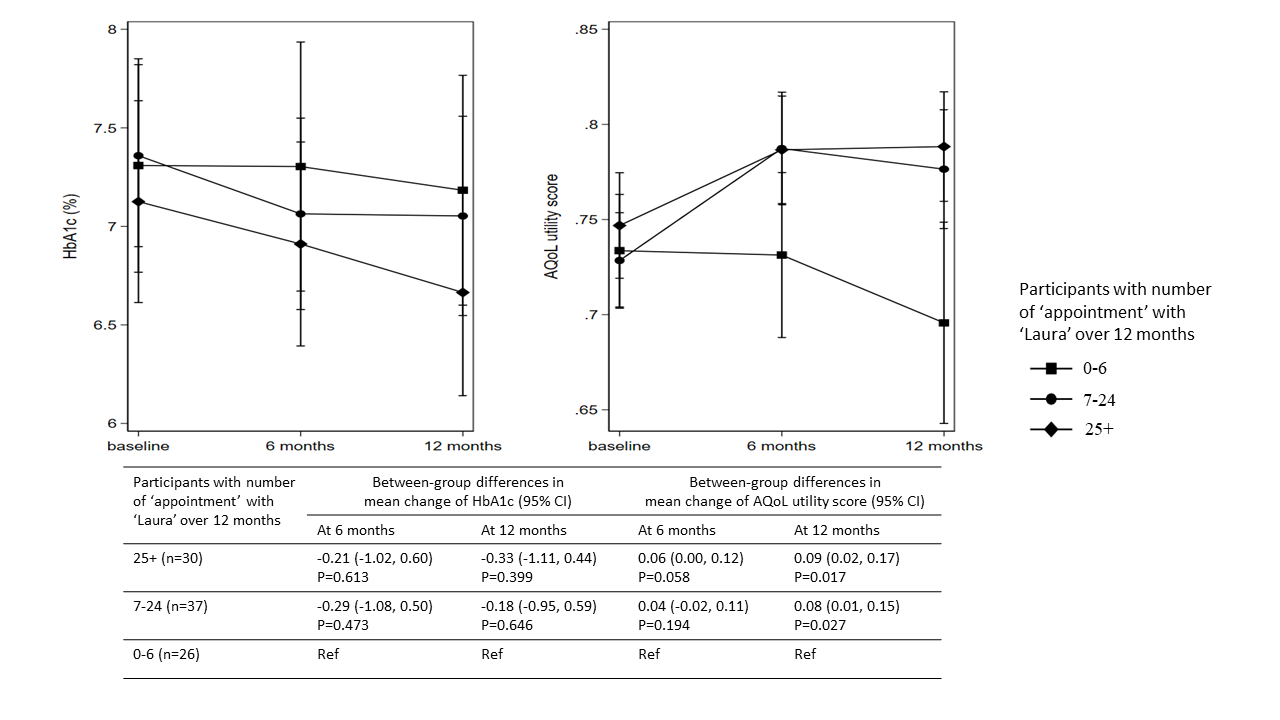


Note: Figures and the table below showed the results from adjusted model where baseline values of variables that are either imbalanced by intervention allocation by chance (baseline age and depression score) or associated with the loss to follow-up (baseline AQoL-8D utility score and HADS Anxiety score).

AQoL: Assessment of Quality of Life
